# The Development of Cognitive Control in Preschoolers and Kindergarteners: The Case of Post-Error Slowing and Delayed Disinhibition

**DOI:** 10.3390/jintelligence12040041

**Published:** 2024-04-01

**Authors:** Maor Yeshua, Andrea Berger

**Affiliations:** 1 Department of Psychology, Ben-Gurion University of the Negev, Beer Sheva 84105, Israel; andrea@bgu.ac.il; 2 School of Brain Sciences and Cognition, Ben-Gurion University of the Negev, Beer Sheva 84105, Israel

**Keywords:** cognitive control, reactive control, proactive control, post-error slowing, delayed disinhibition, intelligence

## Abstract

This study aimed to investigate two specific behavioral manifestations of the executive attention systems in preschoolers and kindergarteners, beyond the unique contribution of intelligence. We tested post-error slowing [${\bar{RT}}_{Post-error\;trial}-{\bar{RT}}_{Not\;post-error\;trial}$] as a marker of reactive control and delayed disinhibition as a novel marker for proactive control. One hundred and eighty preschool- and kindergarten-aged children, as well as their mothers (final sample: 155 children and 174 mothers), performed an adapted task based on Go/NoGo and Stroop-like paradigms—the emotional day-night task. The children showed reliable post-error slowing and delayed disinhibition (mean size effects of 238.18 ms and 58.31 ms, respectively), while the adult size effects were 40–50% smaller. The post-error slowing effect was present for both sexes in all the tested ages, while the delayed disinhibition effect was present only for girls. Both effects showed large individual differences that became smaller in adulthood. Our findings emphasize the earlier maturation of reactive control compared to proactive control, and the earlier maturation of proactive cognitive control in girls compared to boys.

## 1. Introduction

The *executive attention* network is defined as “a set of mechanisms that underlie our awareness of the world and the voluntary regulation of thoughts, feelings, and actions. It involves mechanisms of detection (target detection/error detection), conflict, and cognitive control (inhibition and switching/flexibility)” (Rueda et al. 2023, p. 4). Intertwined with the more general set of executive functions, this attention network enables carrying out goal-directed and flexible actions, and adaptability to changes in the environmental conditions and/or requirements (Blair and Ursache 2011; Rueda et al. 2012, 2023; Welsh and Pennington 1988). Current formulations propose that executive attention actually involves two brain networks, one entailing processes that enable a general maintenance of task performance (i.e., the cingulo-opercular system), and the other entailing processes that enable online response adjustments on a trial-by-trial basis (i.e., the fronto-parietal system) (Dosenbach et al. 2007, 2008; Petersen and Posner 2012; Rueda et al. 2015; Sestieri et al. 2014). The existence of two control brain networks is consistent with the dual mechanism of control framework suggested by Braver (Braver 2012; Braver et al. 2007).

A known critical age period for the development of the executive attention network and cognitive control is early childhood, and specifically, preschool and kindergarten ages (Davidson et al. 2006; Jones et al. 2003; Pozuelos et al. 2014; Rueda et al. 2023; Zelazo et al. 2013; Zelazo and Carlson 2012). Interestingly, the type of processes of executive attention and cognitive control that young children are able to exert might shift from more reactive adjustments on a trial-by-trial basis to more general maintenance of task performance, as a function of age (Chatham et al. 2009; Gonthier et al. 2019; Lucenet and Blaye 2014; Munakata et al. 2012). The current study aims to delve into two specific behavioral manifestations of these systems in preschoolers and kindergarteners.

One well-known behavioral marker of adjustments on a trial-by-trial basis is the post-error slowing (PES) effect (Botvinick et al. 2001; de Mooij et al. 2022). PES refers to the enlarging of the reaction time (RT) after an error, compared to the RT after a correct response (Laming 1979; Rabbitt and Rodgers 1977). Although infants already react to errors, as reflected in their pupil size and brain activity (Berger et al. 2006, 2012; Köster et al. 2019, 2021; etc.), behavioral PES has only been found in children as young as 3 years and 3 months to 5 months while playing “Simon Says”, but not in younger toddlers, as was demonstrated in the pioneering study of Jones et al. (2003). A growing body of work has been exploring the presence of PES in young children (Brewer and Smith 1989; Dubravac et al. 2020; Fairweather 1978; Ger and Roebers 2023a; Gupta et al. 2009; McDermott et al. 2007; and more); however, the developmental course of this effect is far from being well understood. Most studies suggest that PES is more dramatic in young children (i.e., bigger than in adults; Masina et al. 2018; Santesso et al. 2006; Smulders et al. 2016; and more), increasing between age 6 to 7 years (Gupta et al. 2009), or 9 years (de Mooij et al. 2022; Denervaud et al. 2020), and then decreasing to reach the mature/adult size in adolescence. In contrast, Fortenbaugh et al. (2015) found that although PES decreases between childhood and adolescence, afterwards it linearly increases between adolescence and adulthood. These studies did not include children under the age of 6 years. Some studies did have children aged 3–6 years in their sample and found PES (Berwid et al. 2014; Denervaud et al. 2020; Ger and Roebers 2023a); however, they did not analyze the effects of age within their sample. Therefore, one of the aims of the current study is to bridge the gap and test the development of PES within this age group and to be the first study to test age as a continuous variable.

PES can be considered as a marker of behavioral reactive control (i.e., the stimulus-driven elicitation of the control process; Braver 2012). As such, its developmental course could be parallel to what has been learned using the well-known AX-CPT (AX-Continuous Performance Task) paradigm^1^. In a pioneer study, Chatham et al. (2009) showed that 3.5-year-old children exhibited reactive control. Using the same paradigm, Gonthier et al. (2019) and Lucenet and Blaye (2014) pointed out that 5 years old was a critical age for the shift from reactive control to proactive control, which reflects a continuous top-down maintaining of goal-relevant information, and the cognitive top-down flexible adjustments that consider changes in task conditions and requirements (Karayanidis et al. 2011). Therefore, our study explored the development of the PES effect as a marker of reactive control during preschool and kindergarten years. While studying this development, our study took into account additional variables that could explain individual differences in executive control, including fluid intelligence, age and sex, both in adults (Peng et al. 2019; Varriale et al. 2021) and children (Ger and Roebers 2023b; Gómez-Pérez and Calero 2022; Ibbotson and Roque-Gutierrez 2023). Specifically regarding PES, one such variable that correlates with age and could explain part of the variance in PES is fluid intelligence (see meta-analysis by Peng et al. 2019). In adults, fluid intelligence is inversely associated with PES (Varriale et al. 2021). However, the single study that looked for such a correlation in young children did not find empirical evidence for it (Ger and Roebers 2023b). An additional variable of interest in this context is the sex of the child. Although not largely investigated, there are some findings in the literature regarding sex differences in PES. In adults, females show larger PES (Fischer et al. 2016). In children, Torpey et al. (2012) found in a sample of children ranging in age between 5 and 7.5 years, that girls were slower than boys on trials after commission errors, consistent with the overall findings by Else-Quest et al. (2006) that girls tend to be higher in effortful control and lower in impulsivity. Meaning, higher inhibition and executive attention, and lower impulsivity are related behaviorally to greater response time after failed inhibition, as there are greater efforts to prevent it in the near future.

In addition, and in comparison with the development of the PES, the current study also aimed to explore simultaneously the development of a behavioral marker for the second type of control mentioned earlier, that is, the general maintenance of task performance. This type of control was referred to as proactive control in Braver’s (2012) model. It refers to the ability to notice and process wide-context task information and maintain new sets of goal-relevant information (Braver 2012; Regev and Meiran 2014). This type of control has also been studied in terms of the development of cognitive flexibility and task switching (Dajani and Uddin 2015; Moriguchi et al. 2016; Zelazo and Carlson 2012). However, one important aspect that surprisingly has not been studied is the needed flexibility to adapt to changes in inhibition requirements; in other words, the ability to actively disinhibit responses that were previously inhibited (e.g., being able to change the “NoGo” rule by responding to a “Go” stimulus that was defined in the previous block as a “NoGo” stimulus). We suggest referring to this ability as “delayed disinhibition (DD)” and have focused on its development. If DD is a behavioral marker of proactive control, it is possible that the DD effect will change as a function of age in a similar fashion to what has been shown within the AX-CPT task when examining the RT behavioral indicator for proactive control (i.e., ${\bar{RT}}_{AY}-{\bar{RT}}_{BX}$). A relatively linear increase has been found for children of preschool and kindergarten ages (Chatham et al. 2009; Gonthier et al. 2019; Gredebäck et al. 2023; Lucenet and Blaye 2014). Specifically, Chatham et al. (2009) showed that when comparing 3.5-year-old children to 8.5-year-old children, only the older ones show a behavioral manifestation of proactive control. Moreover, as girls tend to outperform boys on executive function tasks, showing greater self-regulation abilities in kindergarten (Matthews et al. 2009) and specifically in cognitive flexibility (Patwardhan et al. 2021), it is possible that the same pattern would be found when exploring the DD effect. Additionally, intelligence was found to be related to proactive control of children in the AX-CPT (Rico-Picó et al. 2021). Therefore, it was important for us to control it when trying to deduce the development of DD. It should be noted that the DD effect complements and expands both the theoretical definition of proactive control (i.e., general maintenance of task information) as well as its operational measurement, as the required control involves maintaining task information through the whole block and is not elicited at the trial level by a precursory cue, as happens in the AX-CPT.

To summarize, our study aimed to investigate two specific behavioral manifestations of the executive attention systems (the PES and DD effects) in preschoolers and kindergarteners, while also assessing the possible moderating effect of sex and controlling for fluid intelligence. Since DD is a more novel control effect, we asked the children’s mothers to perform the task as well, and we analyzed their data for comparison. This additional analysis helped in understanding the behavioral development of the effects, as the mothers’ performance reflected the mature/adult size version of the children’s effects. Regarding the children’s performance, the hypotheses were that: (H1) PES and DD effects would be present in the behavioral responses of preschool and kindergarten children; (H2) the effects of PES and DD would be larger for girls compared to boys; (H3) the effects of PES and DD would increase as a function of age, while PES would behaviorally manifest earlier than DD; (H4) girls’ exact age of behavioral manifestation of cognitive control (both PES and DD effects) would be earlier, compared to boys’ exact age of the behavioral manifestations.

## 2. Method

### 2.1. Participants

An initial sample of 180 preschool- and kindergarten-aged children and their mothers were recruited. Main exclusion criteria, both for mothers and children, were the presence of brain injury or head trauma, as well as neurological disorders (excluding ADHD). Nine children and five mothers did not complete the task and sixteen children and one mother were excluded from the analysis since the correct “Go” rate was lower than 50% (i.e., below chance level). Therefore, the final analyses were done on 155 neurotypical children (79 boys; 51%) and 174 mothers. The children’s ages ranged between 3y-7m to 5y-10m (*M* = 4y-7m ± 5m; girls *M* = 4y-7m ± 5m; boys *M* = 4y-7m ± 6m) and the mothers’ ages ranged between 28y-4m to 51y-6m (*M* = 36y-8m ± 4y-6m).

### 2.2. Ethical Standards and Procedure

The study was approved by the Human Subjects Research Committee of Ben-Gurion University of the Negev, protocol number 2257-1. Ethical standards were followed throughout the study. Recruitment was made via advertisements in social media and the snowball technique (i.e., participants were asked to assist in identifying other potential participants). Mothers signed an online informed consent and the children gave an oral consent upon entry to the lab. Initially, the mothers answered online questionnaires, and afterwards arrived with their child to a meeting in the university campus. In the two-hour meeting, the mothers participated with their children in a battery of tasks. All participants’ anonymity and privacy were ensured. At the end of the lab visit, parents received the equivalent of approximately 33 $ for time and travel expenses and a gift was given to the child.

### 2.3. Measures

The hypotheses of the present paper were tested based on the behavioral scores retrieved from the emotional day-night tasks and the Raven tests. The authors received permission to use these instruments from the copyright holders.

#### 2.3.1. Raven Standard Progressive Matrices (SPM) and Colored Progressive Matrices (CPM) Tests (Adult and Children Raven Tests; Raven 2003; Raven et al. 1998)

The Raven tests includes three sets of 12 figures each, designed to assess fluid intelligence—the CPM in children under the age of 11 years and the SPM in adults. Each figure is a rectangle filled with geometrical shapes and a piece that is missing in a fixed location. The participants are required to choose between six optional complementary parts, with only one part being the correct answer. The final score is the sum of correct responses (0 = *incorrect*; 1 = *correct*) with maximum score of 36 points.

#### 2.3.2. Emotional Day-Night Task (EDNT)

An emotional day-night task was adapted, partially based on Ramon et al. (2011) study, and programmed using E-Prime 2 (the tasks are available at https://github.com/MaorYeshua/EDNT---Children-V.git; Last accessed in 17 January 2024), based on Go/NoGo and Stroop-like paradigms. The participants were exposed to six stimuli: three sun images and three moon images, each with a happy, angry or neutral face (no face was superimposed for the neutral condition). The participants were required to respond to each stimulus via a keyboard button “day” (“P” key) or “night” (“W” key). For children, the task consisted of four blocks, each one composed of 36 trials: 18 day trials (six each of happy, angry and neutral) and 18 night trials (six each of happy, angry and neutral). The first three blocks were congruent, meaning that the child was requested to press a key defined as “day” when a sun was presented, and a key defined as “night” when a moon was presented. The fourth block was an incongruent block, in which these rules were reversed.

**Figure 1:**
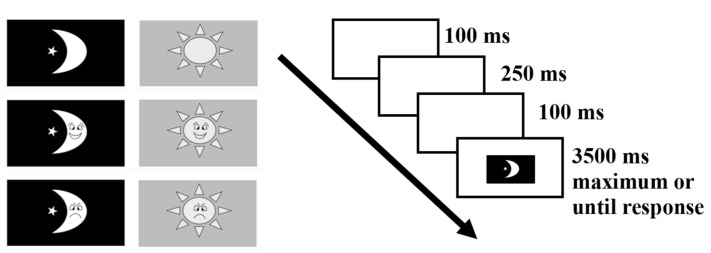
Single flow trial. Sun stimuli were colored in yellow against a blue background. For mothers, maximum appearance time of a stimulus was 2000 ms.

At the beginning of each block, the children were given a “NoGo rule”, meaning that one of the face types (happy/angry/neutral face) was defined as a NoGo stimulus to which they had to inhibit their response. The NoGo stimulus was randomly assigned to each block (see Figure 1 for trial flow and possible stimuli). The adult version of the EDNT was similar to the children’s version in terms of stimuli presented and block rules. However, the adult version consisted of 12 blocks (36 trials each): six congruent and six incongruent. Moreover, the “NoGo rule” was more specific, meaning that one of the six stimuli was defined as a NoGo stimulus.

##### Task Pre-Processing

Since for the first block the definition of DD was irrelevant, it was not included in the analyses. Moreover, the computations of DD and PES effects were conducted on the reaction times for correct Go trials only, meaning that there was a maximum of 72 Go trials for each child and 330 for each mother. According to the minimal RT for visual perceptual stimuli to be processed, if the RT was faster than 150 ms (List et al. 2017) it was removed. Moreover, if it was greater than three standard deviations within each subject, the trial was removed (Matjašič et al. 2018; Ratcliff 1993). After pre-processing of the data, the average trials per child was 53.60 (*SD* = 9.83; 29–70) and per mother was 288 (*SD* = 29; 173–321).

### 2.4. Analytic Plan

The statistical analysis was done using R. The hypotheses H1-H4 (regarding children’s performance) were tested with three nested multiple-regression linear models that were adapted, using DD and PES effects as the dependent variables, and by follow-up analyses of simple slopes.

In order to compute participants’ DD effect, each trial was classified into one of two conditions: (1) *previously inhibited* (PI) if the stimuli was a NoGo in the previous block (i.e., if “angry face” was defined as the NoGo stimulus in block 1, then all the angry face stimuli in block 2 were defined as PI); (2) otherwise, the trial was classified as *not previously inhibited* (NPI). Then, the grand means per participant were subtracted from each other ($\bar{PI}-\bar{NPI}=DD$). In order to compute participants’ PES effect, each trial was also classified into one of two conditions: (1) *post error* (PE) if the previous trial was an error (incorrect Go [i.e., responding incongruently or not responding at all], or an incorrect NoGo trial [i.e., responding to it]); (2) otherwise, the trial was classified as *not post error* (NPE). Then, the grand means per participant were subtracted from each other ($\bar{PE}-\bar{NPE}=PES$). One child had no PE trials, therefore, he was not included in the analysis of the PES effect.

The main analysis between-person variables were (1) sex (0 = *male*, 1 = *female*), (2) age (standardized continuous variable) and (3) their interaction. The first model was an empty model; the second model included sex, age and Raven score; the third model added the two-way interaction terms.

#### Control Variables

As fluid intelligence (i.e., Raven score—standardized continuous variable) increases with age, its main effect and interaction with sex were controlled for in order to explore the unique contribution of age and its interaction with sex to the development of the effects. Moreover, since the children markedly varied in the proportion of correct trials that were previously inhibited (PI) and on the proportion of correct trials that were post error (PE), they were controlled for—PI proportion in the DD effect analysis and PE proportion in the PES analysis (see Table 1 and Table 2 for zero-order correlations). Their proportion from the total amount of correct Go trials was computed (i.e., PI proportion = $\frac{PI\;trials}{N}\;$; PE proportion = $\frac{PE\;trials}{N}$).

An additional control variable that was considered was the socioeconomic status (SES) factor. We found that this factor loaded significantly by mothers’ years of education (.40 loading), household income (.78 loading), number of rooms in the house (.51 loading) and number of family cars (.57 loading). The factor was extracted using the Maximum Likelihood method and the scores were saved based on a regression method. Mothers’ SES factor was separately calculated because of sample size differences, however loadings were highly similar. Since SES did not correlate with DD or PES effects for the children or the mothers (see Table 1 and Table 2), it was not included in the main analyses.

H1 was tested by the intercepts of DD and PES in the empty model. H2 was tested by sex, with DD and PES expected to be larger for girls compared to boys. H3 was tested by age, with DD and PES expected to increase with age. Moreover, region of significance (RoS) analyses would reveal the exact age in which the slopes significantly differed from zero, indicating the behavioral manifestation of them. H4 was tested by the sex x age interaction. The RoS analyses would reveal the exact age in which the slopes significantly differed from zero, with the manifestation of the effects for girls expected to be at an earlier age than for boys.

An additional analysis was conducted on the data of the mothers. The analysis was similar to the one described regarding the analysis of the children’s data; although, the sex variable was irrelevant in the case of the mothers, as all participants were females. As a result, only the first and second model were fitted, without the interaction terms with sex. According to a power calculation conducted using G*power, in order to obtain 80% power, with 5% alpha, two-tailed, with six predictors and a small effect size estimate ($R^{2}=.10$), there was a need for *N* = 132 for the analyses to have sufficient power.

### 2.5. Transparency and Openness

The recruitment and assessment of the children were conducted over a period of two years and all the children were included in the analysis, unless otherwise specified in the Participants section. The current study data is part of bigger study in which Mothers filled a survey built on the Qualtrics platform that included several questionnaires regarding themselves and their child. Afterwards they performed the Raven test, two behavioral tasks that combined Go/NoGo and Stroop-like paradigms (the emotional day-night task and a frustration-inducing Go/NoGo task) and were filmed during two dyadic interactions (the impossible-box task and the etch-a-sketch task). All data for current analyses and the analysis code are available at https://github.com/MaorYeshua/Proacitve-and-Reactive-control---EDN.git; Last accessed in 17 January 2024. The study design and analysis were not pre-registered.

## 3. Results

The results section is ordered according to the study hypotheses. First, we present the findings supporting the existence of the effects in question, and afterwards the main analyses for the main study hypotheses.

### 3.1. Basic Findings

First of all, the reliability of the effects was tested. An odd-even split-half reliability test was performed; for the DD effect, a positive and significant correlation was found (adult version, *r* = .38, *p* < .001, *N* = 174; children version, *r* = .21, *p* = .009, *N* = 155). There was a similar finding regarding the PES effect: adult version, *r* = .48, *p* < .001, *N* = 174; children version, *r* = .27, *p* = .001, *N* = 151.

Second, zero-order correlations were calculated between the study variables for children (see Table 1) as well as for mothers (see Table 2), with Benjamini-Hochberg (BH) correction for false discovery rate. As expected, children who had lower mean RT were older (*r* = −.23, *p* = .022, *N* = 155) as well as with higher Raven scores (*r* = −.34, *p* < .001, *N* = 155). Moreover, children with a greater DD effect had a higher proportion of PI trials (*r* = .32, *p* < .001, *N* = 155), however no such relationship was found for the mothers (*r* = −.11, *p* = .418, *N* = 174). Furthermore, children as well as the mothers with greater PES effect had a lower proportion of PE trials (*r* = −.29, *p* = .003, *N* = 154, and *r* = −.26, *p* = .002, *N* = 174, respectively). An exploratory finding regarding the negative relationship between PES and DD in children was found to be significant (*r* = −.20, *p* = .044, *N* = 154), suggesting a tradeoff between them, while no such tradeoff was found for the mothers (*r* = −.01, *p* = .963, *N* = 174). All of the correlations were also tested using Spearman correlation, and no significant differences between the analyses were found.

### 3.2. Main Analyses

#### 3.2.1. Children

In order to test the study hypotheses, three multiple linear models were calculated for each of the dependent effects, DD and PES, separately, and were fitted as described in the Method section. These analyses were followed-up with simple slopes and RoS analyses. Table 3 presents children analyses. First, the hypotheses testing regarding PES are presented, and then those regarding DD.

Within the empty model of PES, the average PES effect was 238.18 ms, *t*(153) = 7.90, *p* < .001, hence H1 was supported. Predicting PES, the preferable model was the second model, $R_{Adj}^{2}=.088,\;\Delta F\left(4,\;149\right)=4.68,\;p=.001$, using the main effect terms. Within model 2, H2 and H3 were not supported, as no difference was found in sex (β=47.58 ms, *t*(149) = .81, *p* = .417) or age (β=32.58 ms, *t*(149) = 1.05, *p* = .298). H4 was not supported either, as the third model was less preferable.

**Figure 2:**
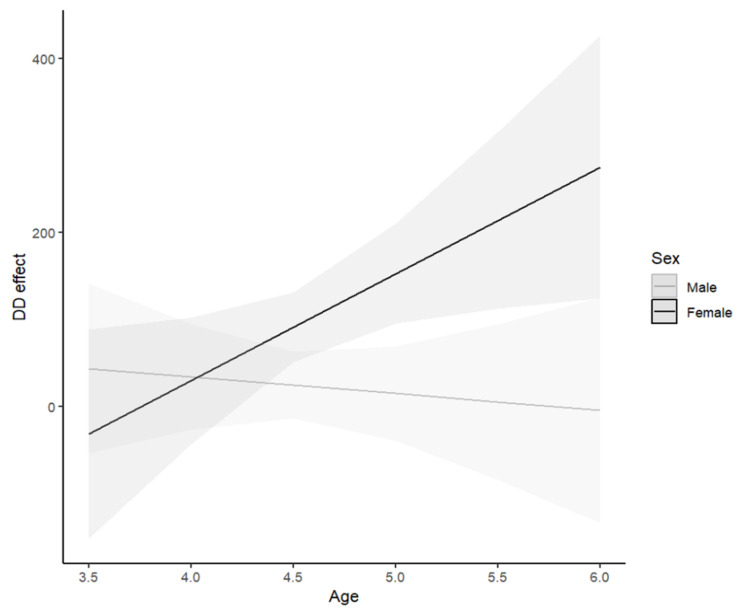
Two-way interaction between sex and age, predicting delayed disinhibition. DD = delayed disinhibition (in milliseconds); age in years; sex represented by lines.

Within the empty model of DD, the average DD effect was 58.31 ms, *t*(154) = 3.92, *p* < .001, hence H1 was supported. Predicting DD, the preferable model was the third model, $R_{Adj}^{2}=.172,\;\Delta R^{2}=.022,\Delta F\left(2,\;148\right)=2.98,\;p=.053$, with the two-way interaction terms. Within model 3, there were sex differences, as girls showed greater DD effect compared to boys (β=75.89 ms, *t*(148) = 2.75, *p* = .007), meaning H2 was supported. The unique contribution of age was not significant (β=−8.43 ms, *t*(148) = −.44, *p* = .659), therefore H3 was not supported. However, there was a significant interaction between sex and age (β=62.62 ms, *t*(148) = 2.09, *p* = .039); see Figure 2.

Simple slopes analysis revealed that for boys there was no relationship between age and the DD effect ($F\left(1,\;148\right)=.20,\;p=.659$), while girls showed a linear increase in the DD effect with age ($F\left(1,\;148\right)=5.47,\;p=.021$). RoS showed that for boys the DD effect size was zero throughout the measured age range (as demonstrated in Figure 2), and for girls the effect began being significant at the age of 4 years and 4 months (DD estimate of 64.08 ms, 95% CI [13.40 ms, 114.70 ms]).

#### 3.2.2. Mothers

The analyses were performed on the mothers as well; see Table 4 for a summary of the findings. Within the empty model of PES, the average PES effect was 138.05 ms, *t*(173) = 20.52, *p* < .001 and within the empty model of DD, the average DD effect was 33.32 ms, *t*(173) = 9.30, *p* < .001, meaning that the PES adult-size effect was 40% smaller than the children’s PES effect size. Moreover, the DD adult-size effect was half compared to the children’s DD effect size. Furthermore, Table 1 and Table 2 indicate a great reduction in PES and DD effect-size variances.

## 4. Discussion

This study aimed to investigate the development of PES and DD effects in preschool and kindergarten boys and girls, beyond the unique contribution of intelligence. Using a computerized task, we were able to test the children’s behavioral manifestation simultaneously, and to determine the exact age of their behavioral manifestation within the tested age period, as well as to compare it to the adult-size effects to learn about their developmental course. The basic analyses demonstrated the validity of the task both in adults and children and supported the importance of taking into consideration the interactions between sex and age, as well as the exact combination of the types of executive control required. We will first discuss the main analysis findings and then the relationship between intelligence, response time, and cognitive control.

### 4.1. Main Findings

Our findings demonstrated that the effect of PES is already present for both sexes at preschool and kindergarten age (3y-7m to 5y-10m). This is consistent with the existing literature that demonstrated this behavioral marker at similar ages (Ger and Roebers 2023a; Gupta et al. 2009; and more) and aligns with Jones et al.’s (2003) pioneering demonstration of behavioral PES, although in a non-computerized task, in a group of children as young as 3y-3m to 3y-5m. At the same time, in our data, the effect of PES did not significantly change in children up to the age of 5y-10m, and there was large variability within the children age group compared to smaller effects and variability of PES within adults (i.e., the mothers). These findings expand the understanding of the developmental trajectory of PES (de Mooij et al. 2022; Gupta et al. 2009), which has been shown to increase with age until around 7–9 years old. As far as we know, the current study is the first to test age as a continuous variable, and it shows that the developmental increase does not occur within our tested age period. Although the lack of difference in our study between boys and girls in PES seems to contradict the findings of Torpey et al. (2012), it should be noted that Torpey et al. found that girls had longer RTs on trials following an error, but they did not compute the PES effect (i.e., the difference in RT between trials following an error and trials following a correct response). Moreover, the electrophysiological data in this study did not show sex differences in the error-related negativity (ERN), which they focused on, and is one of the electrophysiological markers of error processing (Beatty et al. 2020; Botvinick et al. 2001; Coles et al. 2001; Kirschner et al. 2021; and more). The lack of sex differences in the PES that we found within preschool and kindergarten years is consistent with the idea that PES begins developing at ages earlier than 3y-7m (Jones et al. 2003), and is part of the early development of reactive control in general (Chatham et al. 2009). Still, it is reasonable to claim that at this early age this effect is far from being fully developed.

Regarding DD, its presence in the girls of our study from the age of 4y-4m and on is consistent and expands the previous findings by Chatham et al. (2009), Rico-Picó et al. (2021) and others, based on the AX-CPT, that there are aspects of proactive control that are present at earlier ages than 8.5 years. Not only does the effect of DD seem to be present earlier in girls than in boys, but it also keeps developing, as we found a larger effect as a function of age; boys, on the other hand, did not show the effect at all within the age range of our sample. Moreover, as in PES, there was large variability within the children age group, compared to smaller effects and variability of DD within adults (i.e., the mothers). These findings point to a developmental cascade of DD, and align with findings that demonstrated the trajectory of proactive control from childhood to adulthood (Chatham et al. 2009; Gonthier et al. 2016, 2019; Gredebäck et al. 2023; Lorsbach and Reimer 2010; Lucenet and Blaye 2014; Polizzotto et al. 2018; and more). Moreover, they align with the idea that girls’ executive attention network is more developed during this age period (Matthews et al. 2009; Patwardhan et al. 2021). Consistently, girls have also been shown to outperform boys in inhibitory control (Kloo and Sodian 2017), as well as in more general executive functions (see review in Hendry et al. 2016; Matthews et al. 2009) and specifically, in cognitive flexibility (Patwardhan et al. 2021). They are also rated higher in effortful control (Else-Quest et al. 2006) and in self-regulated behavior, and lower in impulsivity (e.g., Blair et al. 2015; Bezdjian et al. 2014; Fuhs et al. 2015; Rea-Sandin et al. 2023). The increase of the DD effect with age for the girls is also in line with the findings that demonstrate a relatively linear increase of proactive control within preschool and kindergarten ages, as measured by the AX-CPT task (Chatham et al. 2009; Gonthier et al. 2019; Gredebäck et al. 2023; Lucenet and Blaye 2014).

The existence of DD only for girls while PES was found for both sexes at preschool and kindergarten age is consistent with claims that the development of reactive control precedes the development of proactive control (Chatham et al. 2009; Rico-Picó et al. 2021). Furthermore, this aligns with the literature claiming that preschool and kindergarten years are a critical age period for the development of the executive attention network and cognitive control (Pozuelos et al. 2014; Rueda et al. 2023). An interesting, but exploratory finding in our study was the tradeoff we found at the individual level between DD and PES, which was not present in the adults (i.e., mothers). At the level of the individual children, larger effects of the general maintenance of task performance (i.e., DD) was accompanied to some extent with smaller effects of online trial-by-trial response adjustments (i.e., PES). Such a tradeoff could imply that within this period of development, when cognitive control is maturing and developing, the children’s limited cognitive control resources might need to be divided between reactive and proactive control. This finding corresponds with findings on typical development across childhood and adolescence that entail changes in the balance between different types of executive control (e.g., Cai et al. 2023; Chevalier et al. 2013). It is possible that such a tradeoff would cease to exist after the full maturation of the cingulo-opercular and fronto-parietal systems, as the findings regarding the mothers demonstrated. Our findings allude to the gradual maturation with age of the cognitive control abilities from kindergarten until adulthood, and probably reflect the increased connectivity within and between the brain networks of executive attention that has been recently demonstrated during this age period (Han et al. 2023). As these are the first developmental findings regarding DD, there is a need for their replication. Moreover, individual differences in cognitive flexibility resulting from life experience (such as active plurilingualism; Bialystok 2015; Morales et al. 2015) might moderate such a relation, as the possible tradeoff between the systems in children at a young age might be related to pre-dispositional differences in adjusting and balancing cognitive control. Future research should be able to replicate these findings with larger samples and with a broader range of ages.

### 4.2. Intelligence

Our findings showed that for preschoolers and kindergarteners, but not adults, fluid intelligence increases with age. Moreover, fluid intelligence predicted faster response times for children and adults. These findings are in line with general findings linking response time and intelligence (Jensen 2006), and are specifically consistent with those of Rico-Picó et al. (2021) that showed greater accuracy and faster response time for children with high intelligence compared to children with middle/low intelligence. Similar findings have also been found in school-aged children (Fry and Hale 2000). However, regarding the relation between intelligence and proactive control within children, our findings contradict those of Rico-Picó et al., as we did not find a correlation between the DD effect and fluid intelligence. This difference could be related to the difference between the specific proactive control markers, and the age ranges in the samples that were tested.

We also did not found a relationship between intelligence and the PES effect within children. This finding corresponds with Ger and Roebers’ (2023b) findings, which also did not find such a correlation for children aged 5–7 years old. Although there is literature indicating a relationship between executive attention in general, and PES specifically, and intelligence (de Mooij et al. 2022; Fortenbaugh et al. 2015; Gupta et al. 2009; Pozuelos et al. 2014; Rothbart et al. 2007; Varriale et al. 2021), the findings are on older children. It is possible that the large variability in the size of the PES effect that we found in our sample prevented the correlation between PES and fluid intelligence to reach significance.

### 4.3. Limitations

The current study has a few limitations that should be mentioned. First, the low reliability of the EDN task which is related to the great within and between subject variability, and the low number of trials in the more difficult conditions, all of which undermine the stability of the measurement. However, as this age period is one of great changes, and the children are very young, these limitations were initially taken into consideration, as a longer task would have resulted in more incomplete data. Moreover, we controlled for the number of trials for each participant in each condition. Another limitation is the absence of measurement of bilingualism. Bilingualism is known to be related to executive control (e.g., Bialystok 2015; Morales et al. 2015). However, it was not measured in the current study. Future research should include such a measure and validate the findings when controlling for it and its interactions with age and intelligence.

## 5. Conclusions

Our findings demonstrated the developmental cascade of PES and DD as behavioral markers of executive attention. During preschool and kindergarten years, children’s limited resources seem to result in a tradeoff between general maintenance of task requirements and the online adjustments needed in real-time, accounting for great variability between children in effect sizes. Such a tradeoff ceases to exist in adulthood as resources grow and the attentional systems mature.

## Figures and Tables

**Table 1:** Zero-Order Correlations between Children Study Variables.

Variables	1.	2.	3.	4.	5.	6.	7.	8.	*M*	*SD*	IQR	Range
1. SES	-								.00	.84	−.50, .68	−2.51, 1.86
2. Sex	.11	-										
3. Age	−.09	−.03	-						4y-7m	5m	4y-3m, 4y-10m	3y-7m, 5y-10m
4. Raven	−.03	−.18 ^+^	.37 ***	-					18.57	4.52	15, 21.50	9, 31
5. PI prop	.10	.04	−.02	.00	-				.43	.08	.36, .49	.25, .60
6. PE prop	.00	.01	−.17	−.22 *	.03	-			.17	.11	.09, .23	.00, .60
7. Mean RT	−.05	.15	−.23 *	−.34 ***	−.03	−.12	-		1676	305.10	1482, 1889	1017, 2737
8. DD effect	.07	.24 *	.04	−.14	.32 ***	.05	.24 *	-	58.31	185.17	−64.48, 176.18	−453.38, 962.97
9. PES effect	.11	.04	.17	.19 ^+^	.03	−.29 **	−.12	−.20 *	238.20	374.13	−15.50, 490.40	−771, 1443.31

*Note. N* = 155/154. SES = socioeconomic status factor, sex (0 = *Male*, 1 = *Female*), DD = delayed disinhibition effect (PI—NPI: PI = previously inhibited, NPI = not previously inhibited), PI prop = proportion of PI trials, PES = post-error slowing effect (PE—NPE: PE = post error, NPE = not post error), PE prop = proportion of PE trials. IQR = inter-quartile range. Mean and standard deviation of mean RT, DD and PES are in milliseconds, age is in years-months format and Raven represents the raw score (the count of correct answers). ^+^ *p* < .1, * *p* < .05, ** *p* < .01, *** *p* < .001, two-tailed, after BH correction.

**Table 2:** Zero-Order Correlations between Mothers Study Variables.

Variables	1.	2.	3.	4.	5.	6.	7.	*M*	*SD*	IQR	Range
1. SES	-							.00	.83	−.47, .67	−2.45, 1.93
2. Age	−.01	-						36y-8m	4y-6m	33y-4m, 39y-9m	28y-4m, 51y-6m
3. Raven	.33 ***	−.10	-					18.18	6.20	14, 22	1, 33
4. PI prop	−.09	.05	−.10	-				.18	.02	.17, .19	.11, .22
5. PE prop	−.06	.01	−.38 ***	.05	-			.11	.06	.07, .14	.02, .36
6. Mean RT	−.14	−.01	−.34 ***	.08	.16	-		689.90	118.11	605.50, 768.50	472, 1073.40
7. DD effect	.12	−.06	.02	−.11	−.12	.03	-	33.32	47.25	1.40, 58.23	−89.68, 180.33
8. PES effect	−.10	−.04	.01	.00	−.26 **	.44 ***	−.01	138.05	88.74	76.87, 188.75	−27.14, 504.26

*Note. N* = 174. SES = socioeconomic status factor, DD = delayed disinhibition effect (PI—NPI: PI = previously inhibited, NPI = not previously inhibited), PI prop = proportion of PI trials, PES = post-error slowing effect (PE—NPE: PE = post error, NPE = not post error), PE prop = proportion of PE trials. IQR = inter-quartile range. Mean and standard deviation of mean RT, DD and PES are in milliseconds, age is in years-months format and Raven represents the raw score (the count of correct answers). ** *p* < .01, *** *p* < .001, two-tailed, after BH correction.

**Table 3:** Children’ Multiple Linear Models, with Post-Error Slowing and Delayed Disinhibition as Dependent Variables.

		Post-Error Slowing								
Model Effects		Model 1			Model 2			Model 3		
		Estimate	*SE*	*p* Value	Estimate	*SE*	*p* Value	Estimate	*SE*	*p* Value
	Intercept	238.18	30.15	<.001	216.70	40.72	<.001	217.64	40.97	<.001
	PE proportion				−94.50	29.74	.002	−97.90	29.96	.001
	Raven score				45.16	32.55	.167	36.82	41.44	.376
	Sex (0 = *male*; 1 = *female*)				47.58	58.51	.417	49.07	58.74	.405
	Age				32.58	31.17	.298	62.51	40.51	.125
	Sex × Raven score							27.74	66.49	.677
	Sex × age							−74.53	64.02	.246
	Adjusted *R*^2^				.088			.084		
	*F*				4.70 ***			3.35 **		
	Δ *R* ^2^							.000		
	Δ *F*				4.68 ***			.68		
		Delayed Disinhibition								
	Intercept	58.31	14.88	<.001	21.28	19.39	.274	22.93	19.24	.235
	PI proportion				57.73	13.77	<.001	53.25	13.84	<.001
	Raven score				−26.04	15.09	.086	−32.31	18.83	.088
	Sex (0 = *male*; 1 = *female*)				75.51	27.96	.008	75.87	27.64	.007
	Age				18.71	14.84	.209	−8.43	19.07	.659
	Sex × Raven score							11.08	31.28	.724
	Sex × age							62.62	30.01	.039
	Adjusted *R*^2^				.150			.172		
	*F*				7.78 ***			6.32 ***		
	Δ *R* ^2^							.022		
	Δ *F*				7.99 ***			2.98 *		

*Note. N* = 154/155; PE = post-error stimuli; PI = previously inhibited stimuli; age and Raven scores are standardized. Estimates are in milliseconds. * *p* < .05; ** *p* <. 01; *** *p* < .001.

**Table 4:** Mothers’ Multiple Linear Models, with Post-Error Slowing and Delayed Disinhibition as Dependent Variables.

		Post-Error Slowing					
Model Effects		Model 1			Model 2		
		Estimate	*SE*	*p* Value	Estimate	*SE*	*p* Value
	Intercept	138.05	6.73	<.001	138.05	6.50	<.001
	PE proportion				−27.19	7.07	<.001
	Raven score				−9.80	7.10	.170
	Age				−4.15	6.56	.528
	Adjusted *R*^2^				.065		
	*F*				5.02 **		
		Delayed Disinhibition					
	Intercept	33.32	3.58	<.001	33.32	3.58	<.001
	PI proportion				−4.96	3.62	.172
	Raven score				.04	3.63	.992
	Age				−2.55	3.62	.482
	Adjusted *R*^2^				.00		
	*F*				.84		

*Note. N* = 174; PE = post-error stimuli; PI = previously inhibited stimuli; age and Raven scores are standardized. Estimates are in milliseconds. ** *p* < .01.

## Data Availability

The data and task are available at https://github.com/MaorYeshua/EDNT---Children-V.git (Last accessed in 17 January 2024).

## Note

1 In this task there is a cue (usually A or B) followed by a probe (usually X or Y). The participants are asked to respond with one finger to a target stimulus that is more frequent than the other combinations (the AX sequence) and with another finger to all non-target stimuli (AY, BX, and BY sequences). One of the proactive control behavioral markers is the higher RTs in AY trials (as A is a contextual information cueing for the appearance of X) and lower RTs in the BX trials (as B is a contextual information cueing for the trial to be classified as a non-target, hence the appearance of X generates less delay in RT).
